# Functional near-infrared spectroscopy: a continuous wave type based system for human frontal lobe studies

**DOI:** 10.17179/excli2015-614

**Published:** 2015-10-23

**Authors:** Sigita Venclove, Algis Daktariunas, Osvaldas Ruksenas

**Affiliations:** 1 Department of Neurobiology and Biophysics, Faculty of Natural Sciences, Vilnius University, Ciurlionio 21/27, LT-03101 Vilnius, Lithuania

**Keywords:** functional near-infrared spectroscopy, non-invasive, hemodynamic response, cortical activation, prototype

## Abstract

Functional Near-Infrared Spectroscopy (fNIRS) is an optical non-invasive brain monitoring technology that registers changes in hemodynamic responses within the cortex of the human brain. Over the last decades fNIRS became a promising method in neurosciences: it is non-invasive, portable and can be used in long term studies. All these advantages make it suitable for educational purposes as well. This paper presents basic methodological concept of optical engineering principles and suitable applications of fNIRS. We represent a continuous wave (cw-fNIRS) system that could be used for frontal lobe studies in human adults or as demonstration equipment for physiological measurements. This system has been validated by comparing it with commercial device fNIR400 from Biopac. A comparison of geometry, data and statistical analyses suggests similar hemodynamic responses recorded by both devices. Our study suggests that this system can be used for further development and as a guideline for researchers to develop a specific tool for applications in human brain studies.

## Introduction

Functional Near-Infrared Spectroscopy (fNIRS) is an optical brain spectroscopy and imaging method that enables continuous, non-invasive and portable monitoring of changes in blood oxygenation and blood volume related to human brain functions. fNIRS has become a common method because of several advantages: it is relatively inexpensive; non-invasive; has good both space and time resolution (about 1 s and 1 cm^2^) (Strangman et al., 2002[15]) and can be used repeatedly or constantly with no adverse effects (Ferrari and Quaresima, 2012[6]). The application of fNIRS in cerebral functioning studies has been validated by other neuroimaging techniques, showing that the data maintains a strong correlation with data from PET and fMRI BOLD method (Huppert et al., 2006[8]). However, for experiments it's still relatively little used. The main reason may be the lack of practical knowledge in its application, confusion about its pros and cons in general, and costly together with fixed-use commercial devices. 

The main advantages of custom made system would be: 

a) low cost (we used only common and globally available components), 

b) easy to make (requires only basic knowledge of electronics) and 

c) ease of modification (i.e. to specific layout of sensor geometry). 

All this led us to development of a non-commercial system that would be suitable for use in our laboratory and at the same time would be an example for students, independent investigators and teachers searching examples for personalized biological data acquisition equipment.

**Figure 1:**
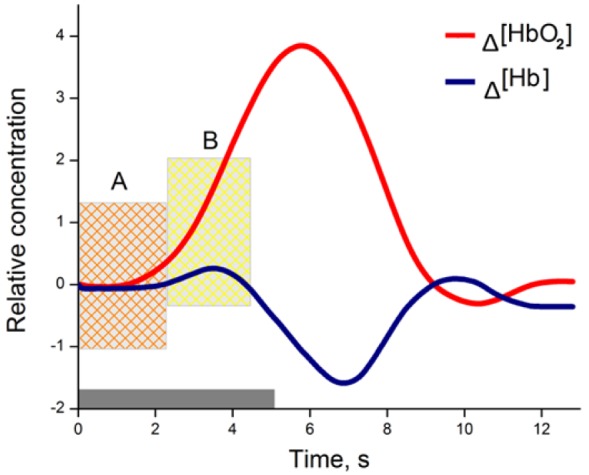
Changes in hemoglobin concentration due to neural activity with stimulation from 0 to 5 seconds (grey bar); A- inflow of oxy-Hb, when total blood volume is relatively unchanged; B- oxy-Hb rises rapidly during vasodilatation as well as total blood volume.

Brain activity is associated with a number of physiological processes, in particular to neurovascular coupling, which can be assessed in the optical window of 650-950 nm light. This optical window allows light to penetrate deep enough through the skin and skull to the surface of the human brain. During neural activity, physiological processes like hemodynamic response (HR) can be detected and measured using optical techniques (Figure 1(Fig. 1)).

Most biological tissues are relatively transparent to light in the near-infrared range, but chromophores like oxy-Hb, deoxy-Hb or cytochrome-c-oxidase absorb a fair amount of energy. During spectroscopy, changes of light absorbance reveal relative local changes in chromophore concentration which indicates increased neural activity in the specific brain area (Strangman et al., 2002[15]).

Hemodynamic responses associated with the brain activity can be measured as concentration changes (µM), hemoglobin signal (mM*mm) or absolute changes in amplitude which is the most common. The two main groups of used amplitudes are concentrations of chromophores (mainly Δ[HbO_2_], Δ[Hb]) and absolute response parameters (mainly blood volume Δ[Hb_total_] and blood oxygenation change Δ[O_2_]).

The mathematical basis of near-infrared spectroscopy is a modified Beer-Lambert law (MBLL) (Kocsis et al., 2006[10]). The differential form of MBLL (dMBLL) states that the change in light attenuation is proportional to the changes in the concentrations of chromophores in tissue, mainly oxy-Hb and deoxy-Hb (Kocsis et al., 2006[10]):


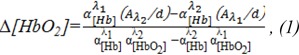



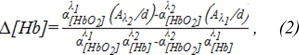


where: α is the specific absorption coefficient for a given tissue ([molar^-^^1^, mm^-^^1^]); A- optical density or absorbance at the wavelength λ and thickness of sample d. Total change of oxygen is defined as


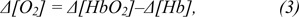


and total blood volume as


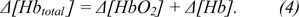
.

Beginning with the pioneering work of Jöbsis (1977[9]) when non-invasive NIRS was first utilized to experimentally investigate cerebral oxygenation, multichannel NIRS instruments have been widely applied to examine the functional activation of the human cerebral cortex in adults and in newborns (Wolf et al., 2007[17]).

Because of rich nature of a hemodynamic signal (multiple components often defined as noise (Bauernfeind et al., 2014[1];Tong et al., 2011[16]) fNIRS systems are used with other physiological and neurobehavioral measures such as the heart rate variability and respiration (Durantin et al., 2014[4]). Furthermore, relatively low cost devices accelerate data acquisition and accumulation processes for benefits such as novel development of a brain-computer interface (BCI) (Coyle et al., 2007[3]). 

The main fields, where fNIRS has been applied since it was introduced are listed in Table 1(Tab. 1). Information was collected searching with keywords (listed in Table 1(Tab. 1)) in ScienceDirect database.

fNIRS can be used to evaluate brain activation related to cognitive tasks and performance during normal daily activities. This potential exists more for fNIRS than for any other neuroimaging modality (Ferrari and Quaresima, 2012[6]; Wolf et al., 2007[17]). The fNIRS technique has various names such as Diffuse Optical Tomography (DOT) or Near-Infrared Imaging (NIRI), depending on the circumstances and the diversity of techniques that can be used.

There are various modifications possible due to the relatively simple design. The three dominating types are:

1. continuous wave (CW) - based on constant tissue illumination, simply measures light attenuation through the head;
2. frequency-domain (FD) - illuminating the head with intensity-modulated light, measures both attenuation and phase delay of emerging light;
3. time-domain (TD) - detects the shape of the short pulse after propagation through tissues (Mansouri and Kashou, 2012[12]).

However, the continuous wave type devices are the most common. There are several CW-fNIRS systems and sensor designs that allow this technology to be used for infants as well as adults, under stationary or portable conditions (León-Carrión and León-Domínguez, 2012[11]; Homae et al., 2011[7]). CW-fNIRS is characterized by the simplest equipment, data acquisition and calculations.

As was mentioned above, the main advantages of fNIRS as compared to other neuroimaging modalities are following - ease of administration (i.e. no special requirement for surrounding and exploitation as is with fMRI) and direct measurement of both Δ[HbO_2_] and Δ[Hb] concentrations.

To sum up, NIRS is a more convenient and less expensive technology than other neuroimaging methods.

## Methods

### Instrumentation

Our goal was to design CW-fNIRS system that could be used for frontal lobe studies in human adults and could also be easily replicated and expanded for any needs. To meet this goal we created a system design which could be a system for one to two optodes in the early stage but flexible for multichannel probes in future usage.

The proposed system is built of four primary components:

1. a forehead sensor,
2. a sensor control unit for synchronization and initial data processing,
3. a data acquisition module (DAQ) for further data processing and
4. a laptop computer with data acquisition and calculation software.

The developed sensor consists of two LED light sources and two detectors, with a source-detector separation of 2.5 cm. Sensor configuration can be described as O X X O, where O is the detector, and X is the source. The light sources have wavelengths of 735 nm (RED) and 850 nm (IR). The photodetectors are monolithic photodiodes with a single supply transimpedance amplifier. The sensor design is flexible and allows for the attachment of the sensor to the subject's head. However, O X pairs or optodes are immobilized to prevent major movement artifacts. As a background we used polymeric clay which is easy to acquire and to make steady but flexible form. 

**Figure 2:**
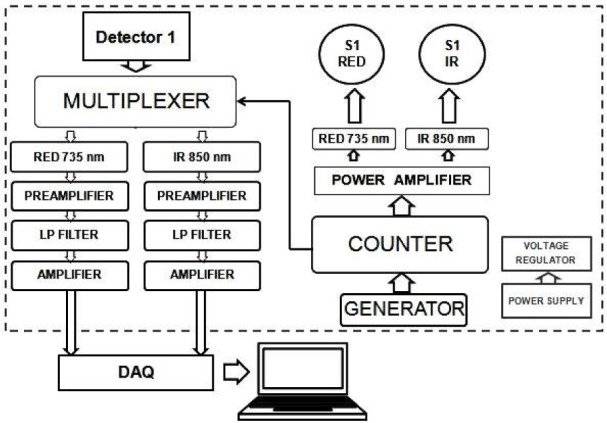
Simplified block diagram of one optode sensor control unit. One light source consists of two wavelengths (850 IR and 735 RED). By the counter they are turned on one by one in the sequence. At the same time, counter triggers multiplexer to sample it from the detector. Both samples are amplified, filtered from noise and transmitted to DAQ separately. Thus one optode is a pair of source and detector, and one optode has two channels. DAQ-data acquisition module, S1-Source number 1, LP- low-pass filter.

The sensor control unit ensures synchronization between the emitter and the detector, the initial data recording and transmission to the DAQ (see Figure 2(Fig. 2)). A simplified block diagram of the one channel sensor control unit shows how the emitter and detector work is synchronized and channel light sources are sampled at 300 µs intervals. Signals coming from registering photodetectors are divided into separate channels, filtered with a low pass filter (∽10 Hz) and amplified. Using data acquisition module information is sent to and stored on the PC.

An offline signal processing algorithm digitally filters raw data file and calculates absolute Δ[HbO_2_], Δ[Hb] changes, blood volume Δ[Hb_total_] and oxygenation change Δ[O_2_] after the experiment. Data of each parameter are saved separately into text files and visualized in the form of graphics.

In order to validate the results of our system we repeated tests with a commercial CW-type 16 optode device fNIR400 from Biopac. This sensor has four light sources and ten detectors with the same source-detector separation of 2.5 cm. It also has similar LED wavelengths of 730 nm and 850 nm. Due to very low sampling frequency (2 Hz) this device records only major hemodynamic response component. 

**Figure 3:**
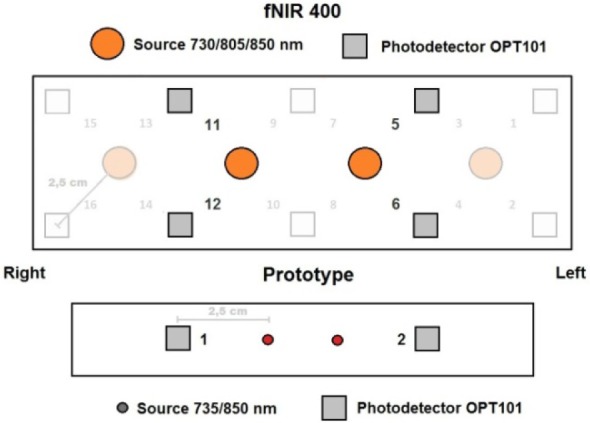
Commercial (top) and proposed system (bottom) sensor geometry and central alignment according to the 10-20 EEG system. Relevant optodes are highlighted.

Due to the differences in sensor geometry, we made a comparison of fNIR400 and system optodes: 1^st^ and 2^nd ^system optodes correspond to 5^th^, 6^th^ and 11^th^, 12^th^ of fNIR400 (see Figure 3(Fig. 3)). Note that both sensors on human head were placed around Fp1 and Fp2 according to the 10-20 EEG system. Both sensors were aligned according to pre-manufactured fNIR400 sensor central marks.

Processing of data and comparison of results from both devices were conducted using different software: acquisition and processing was conducted with MATLAB for our system and pre-installed fnirSoft software for fNIR400; for data comparison was used MATLAB from *MathWorks* and Origin Pro from *OriginLab*. Note that, if data is saved in *.txt* format files, it can be also processed with open source scripting software analogs such as Python.

Further comparison of the main technical characteristics of both devices is presented in Table 2(Tab. 2).

### Experiments

This experiment was approved by the ethical committee of the Regional biomedical research ethics committee in Vilnius. All subjects were informed with written consent and agreed to participate in this research voluntarily. To justify that designed system recorded hemodynamic response, the pilot experiments were performed with a validated and tested in clinical studies Wisconsin Card Sorting Test (WCST) (Fallgatter and Strik, 1998[5]; Cianchetti et al., 2005[2]; Nyhus and Barceló, 2009[14]). This task significantly activates frontal lobes during the all performance time. Furthermore, WCST is also used to diagnose frontal lobe disorders (Nyhus and Barceló, 2009[14]). This task was used only to evoke HR, and no conclusions were made about the quality of the subject's performance.

The WCST was performed using the same protocol for both devices:

a) Introducing the task and giving instructions; 

b) Recording baseline with closed eyes in calm position (-60 s for system and -10 s for fNIR400);

c) Recording the experiment (up to 300 s). 

Recording baseline length is different because fNIR 400 recordings were made with built-time duration. In the last stage the subject's performance time was marked individually (the rest of the time subject was asked to stay calm and with closed eyes until experiment ends in order to measure the reverse of chromophores concentration after WCST). 

For testing constructed system or for preparing educational demonstrations the researchers can use free version of Wisconsin Card Sorting Test WCST also known as Berg Card Sorting Test (BCST) from The Psychology Experiment Building Language (PEBL) and Test Battery (Mueller and Piper, 2014[13]).

.

## Results

### Developed system

**Figure 4:**
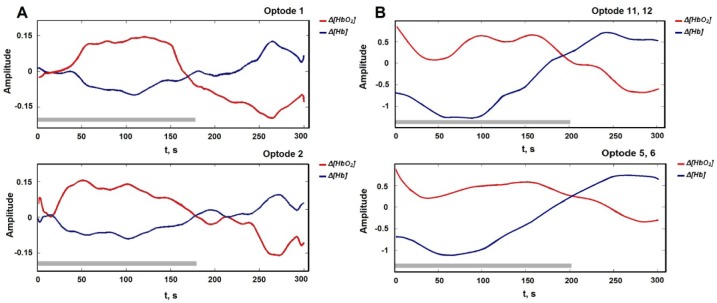
(A) Averaged fNIRS signals from developed system for each channel: Δ[HbO_2_] quickly rises during the WCST and slowly drops after; grey bar - averaged task time (~178 s); averaged response time with SD: HR_Op1_= 181.12 ± 18.92 s and HR_Op2_= 182.5 ± 18.43 s. (B) Averaged fNIRS signals from fNIR400: grey bar indicates average task time (~197 s) and averaged hemodynamic response time: HR _Op 11, 12_= 199.12 ± 15.85s and HR _Op 5, 6_= 204.02 ± 15.34 s.

The WCST was performed with eight 22 ± 2 year old male subjects. Strong negative correlation between the recorded Δ[HbO2] and Δ[Hb] signals (r> -0.90) shows that the concentration of chromophores differs from each other during the WCST (Figure 4(Fig. 4)). It also can be seen as evidence for real signal detection. A paired t-test revealed that parameters of hemodynamic response (Δ[HbO2] and Δ[Hb]) differ with a significance level of p= .02. Data from both channels show that the average task performance time and standard deviation were similar but not identical. Hemodynamic response differences recorded from optodes one and two are shown in Figure 4(Fig. 4). In eight subjects range of concentration variation was higher in Δ[HbO2] (0.13) than Δ[Hb] (0.06) as expected. 

### fNIR400

The WCST was repeated with eleven 24 ± 3 year old male subjects. For further analysis from all 16 optodes we included optodes 11^th^ and 12^th ^according to system optode 1 (right hemisphere) and optodes 5^th^ and 6^th^ for optode 2 (left hemisphere, see Figure 3(Fig. 3)). Averaged data from right-handed males showed that there is the same HR delay in the time for left hemisphere in fNIR400 as it was with developed system (Figure 4(Fig. 4)). Furthermore, using paired t-test we found that Δ[HbO_2_] and Δ[Hb] concentration was higher in the left optodes 5 and 6 than in the right optodes 11 and 12 with significance *p< .5 *(Table 3(Tab. 3)). However, ratio of concentration variability was similar to developed system: Δ[HbO_2_] (3.0) and Δ[Hb] (1,6).

### Both devices

Comparison of data measured with developed system and commercial device revealed similar characteristics of the HR signal. These results suggest that developed CW-fNIRS system could be used for the frontal lobe studies in human adults. Furthermore, results indicate small but measurable differences of hemispheric activation. Because this difference was detected in both cases, it should depend on the selected task, but not random variation. A brief comparison of both experiments is shown in Table 4(Tab. 4).

## Discussion

Our goal was to develop fNIRS system that would be suitable for various neuroscientific studies and education. Given the fact that it can be created by non-professional engineers and all elements are available in the global market, we believe that our design can be easily adapted to any researchers needs without any special requirements and based on the main knowledge of electronics. According to preliminary calculations a similar device can be made for less than 80 USD or EUR when ordered equipment is considerably more expensive.

We assume that there were no reason to associate received hemodynamic response with artifacts such as blood flow changes in human forehead skin or random optical signal fluctuations, as the resulting signals have a good separability from noise and each other (Δ[HbO_2_] from Δ[Hb]) and also correlate with presentation of stimulus.

Nevertheless, our study can be improved by addressing these concerns: the effects of motion artifacts were minimized by giving subjects instructions to reduce their activity to minimal during the experiment. This could be improved with parallel motion measurement using accelerometer and subtracting it from optical signal directly. The locations of measurements with both devices were superposed, but still are not equal. Therefore, the comparison of the system and fNIR400 should be repeated after increasing the number of system channels. 

It should be noted that proposed system is not officially certified with CE as manufactured devices and researchers should rebuild and use it with their own caution. However, proposed system is not harmful and corresponds to electrical safety standards for medical equipment.

Finally, we expect that our paper will fulfill the lack of simple methodological information about how to build a simple functional near infrared spectroscopy device for any individual needs. 

## Conclusions

A continuous wave fNIRS system designed in our Department registers frontal lobe activity during cognitive task performance as hemodynamic response and can be used in further studies or development. Our device is designed for individual development purposes and could be relatively inexpensive to build with a specific number of channels and optodes geometry for many studies.

## Conflict of interest

The authors declare that they have no conflict of interest.

## Figures and Tables

**Table 1:**
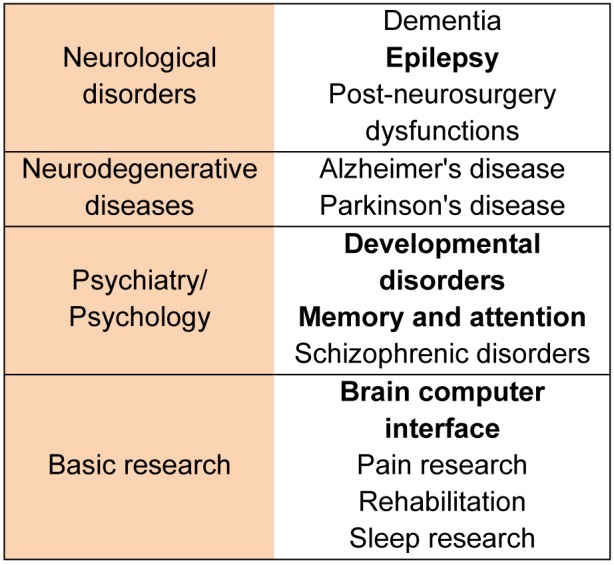
The most popular fNIRS applications in neuroscience. Fields with more than 100 results in *ScienceDirect* are bold.

**Table 2:**
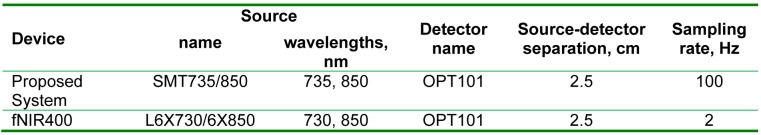
The main parameters of proposed and commercial fNIRS devices

**Table 3:**
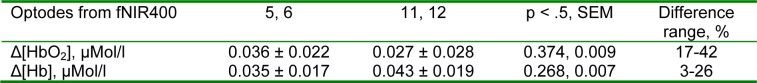
Commercial fNIRS device data comparison. Significance between pairs *p< .5. *N=11

**Table 4:**
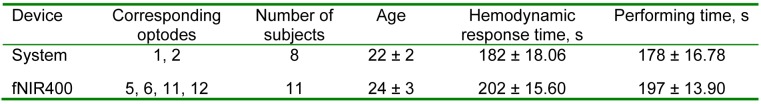
Data collected from developed system and commercial fNIRS devices, mean ± SD
